# MMP-9 expression varies according to molecular subtypes of breast cancer

**DOI:** 10.1186/1471-2407-14-609

**Published:** 2014-08-23

**Authors:** Einas M Yousef, Muhammad R Tahir, Yves St-Pierre, Louis A Gaboury

**Affiliations:** Institute for Research in Immunology and Cancer, Université de Montréal, Montréal, Canada; Le centre de Recherche du Centre Hospitalier de l’Université de Montréal, Montréal, Canada; INRS-Armand-Frappier, Université du Québec, Laval, Montréal, Canada; Department of Pathology and Cell Biology, Faculty of Medicine, Université de Montréal, Montréal, Canada

**Keywords:** MMP-9, Human breast cancers, Metastasis, *In silico* analysis, Tissue microarrays

## Abstract

**Background:**

In 2014, breast cancer remains a major cause of mortality worldwide mostly due to tumor relapse and metastasis. There is currently a great interest in identifying cancer biomarkers and signalling pathways mechanistically related to breast cancer progression. Matrix metalloproteinase-9 (MMP-9) is a member of matrix degrading enzymes involved in cancer development, invasion and metastasis. Our objective was to investigate MMP-9 expression in normal human breast tissue and to compare it to that of breast cancer of various histological grades and molecular subtypes. We also sought to correlate MMP-9 expression with the incidence of metastasis, survival rates and relapse in breast cancer patients.

**Methods:**

MMP-9 was first studied using *in silico* analysis on available DNA microarray and RNA sequencing data of human breast cancer tissues and human breast cancer cell lines. We next ascertained MMP-9 expression in both normal breast tissue and in human breast carcinoma tissue microarrays.

**Results:**

Significant increase in MMP-9 expression was found in breast cancer cells where compared to normal breast tissue. A positive correlation could also be established between elevated levels of MMP-9 and breast cancer of high histological grade. Furthermore, our results indicate that not only MMP-9 is differentially expressed between each molecular subset but also, more importantly MMP-9 overexpression revealed itself as a startling feature of triple-negative and HER2-positive breast cancers. Lastly, the clinical relevance of MMP-9 overexpression is strongly supported by its significant association with a higher incidence of metastasis and relapse.

**Conclusions:**

Differential expression of MMP-9 reflects the extent of cellular differentiation in breast cancer cells and is closely related to the most aggressive subtypes of breast cancer. Hence, MMP-9 is a promising prognostic biomarker of high-grade breast cancer. In our opinion, MMP-9 expression could help segregate subsets of aggressive breast cancer into clinically meaningful subtypes.

**Electronic supplementary material:**

The online version of this article (doi:10.1186/1471-2407-14-609) contains supplementary material, which is available to authorized users.

## Background

Breast cancer is the most common malignancy and the second leading cause of cancer-related death after lung cancer among women in the United States and Europe [1]. Due to major advances in screening and early diagnostic procedures, most breast cancer patients are diagnosed at an early stage. However, 6% to 10% of patients still present with metastatic breast cancer at the time of diagnosis; for those patients, relapses tend to occur earlier and survival rates are shortened [2]. Cancer metastasis is considered to develop in a step-wise fashion leading to the acquisition of new capabilities by tumor cells helping them to thrive and evade natural barriers [3]. Cancer cells detach themselves from the primary tumor, migrate and invade surrounding tissues, enter the vasculature, circulate throughout the body and eventually reach secondary sites where they extravasate, and populate distant organs [4].

Degradation of the extracellular matrix (ECM) is thought to be a crucial step in the formation of tumor metastasis. Multiple proteolytic enzymes such as plasmin, cathepsins, and matrix metalloproteinases (MMPs) are known to degrade ECM [5]. Matrix metalloproteinase-9 (MMP-9) is a zinc-dependent peptidase that belongs to the gelatinase subfamily of MMPs. It is excreted as an inactive pro-enzyme that undergoes activation upon cleavage by different types of extracellular proteases [6]. MMP-9 activity is thought to be regulated by different biochemical stimulators such as growth factors and cytokines whose expression appear to modulate intracellular signaling pathways [7]. MMP-9 has the ability to degrade denaturated collagens which have been first cleaved by various collagenases such as MMP-1, MMP-8 and MMP-13 [8, 9]. In addition, MMP-9 degrades type IV collagen which is the main component of the basement membrane [10]. It exerts different roles in the dissemination process such as tumor invasion, tumor-induced angiogenesis, and immunomodulation of the tumor microenvironment. In addition, MMP-9 is instrumental in creating so-called premetastatic niches that foster colonization of distant organs [11]. Elevated tissue levels of MMP-9 are also associated with invasion, metastasis and poor prognosis in different types of cancer including cervical [12], colorectal [13], ovarian [14] and breast cancer [15]. Furthermore elevated levels of MMP-9 in the serum and urine have also been found to be associated with metastasis and poor prognosis in a diversity of cancers [16].

Our goal was to assess the potential clinical usefulness of MMP-9 as a prognostic biomarker of breast cancer. To achieve that aim, we first studied *MMP*-*9* mRNA expression using *in silico* analysis on available DNA microarray and RNA sequencing data of human breast cancer tissues and breast cancer cell lines. We next evaluated MMP-9 expression at the protein level using immunohistochemical analyses on tissue microarrays containing both normal and neoplastic breast tissues. Our data were next correlated with patients’ outcome specifically looking at the incidence of metastases, relapse and overall survival. Our results indicate that MMP-9 is not only differentially expressed in different molecular breast cancer subtypes but also overexpressed in triple-negative and HER2-positive breast cancers. Overexpression of MMP-9 tightly correlates with a higher incidence of metastasis and relapse. Taken together, our data indicate that differential expression of MMP-9 reflects the degree of differentiation of breast cancer cells and that its overexpression tightly correlates with the most aggressive subtypes of breast cancers. Hence, MMP-9 is a potentially useful biomarker of aggressive and metastatic subtypes of breast cancer.

## Methods

### *In silico*analysis

The web application bc-GenExMiner [17] was used for correlation analysis of MMP-9 gene expression on a dataset comprising over 3,063 microarrays. However, only 1210 patients could be correctly assigned precisely to each molecular subtype. The “aov” and “TukeyHSD” functions were carried out to compare the mRNA levels within each breast cancer molecular subtypes. The ANOVA was applied to check for an overall difference of expression levels between each molecular subtypes. The Tukey multiple comparisons of means were used to test for a significant difference between two subtypes (e.g. Luminal A vs. Basal). For both tests, a p-value < 0.05 was considered significant. The mRNA level of MMP-9 in 51 breast cancer cell lines were also studied using publically available microarrays and mRNA sequencing breast cancer cell line datasets [18].

### Patients and tissue samples

A retrospective study was carried out using a cohort of 300 female breast cancer patients comprising tumors of different histological grades. Archived Formalin-fixed, paraffin-embedded (FFPE) samples containing tumor tissues were collected for the study. Tumor grades were confirmed using the Modified Scarff-Bloom-Richardson-Elston-Ellis grading system (SBR-EE) [19]. A complete set of follow-up data including the onset of metastasis and relapse were acquired. We also obtained 19 normal breast tissues from healthy women undergoing plastic surgery to serve as internal controls. Benign breast conditions such as mammary fibroadenoma and myofibroblastoma were included as negative controls [20]. In addition, a number of extraneous tissues such as colon, thyroid and placenta were included in each TMA. All samples were obtained from *Centre Hospitalier de l*’*Université de Montréal* (CHUM) after granting the approval of the research ethical committee (Comité d'éthique de la recherche du CHUM CENTRE DE RECHERCHE, Approval No. SL 05.019).

### Tissue microarray (TMA)

Sections (4 μm) from each paraffin block were stained with hematoxylin and eosin (H&E) and examined by two independent pathologists. Core punches, 1 mm in diameter, were drilled from representative areas contained within each FFPE tumor blocks. Each core was realigned in duplicate or triplicate into recipient blocks according to the intended design of the map using a Manual Tissue Arrayer I (Beecher Instruments). Blocks were next inverted and incubated overnight in the oven over a glass slide. TMA blocks were allowed to cool until they could easily detach from the glass slide. Tissue sections from each TMA were prepared and one slide from each block was stained with H&E to review the diagnoses and histological grades on all tissue samples. Additional representative sections from each block were submitted to automated immunohistochemical (IHC) staining.

### Immunohistochemistry

Immunohistochemical assays were performed on FFPE tissues obtained from each TMAs. These assays were carried out according to manufacturer recommendations on an automated immunostainer (Discovery XT system, Ventana Medical Systems, Tucson, AZ). Immunohistochemical analysis of MMP-9 (polyclonal; ab38898, dilution 1/100, no pretreatment, Abcam, Canada) was carried out to detect both the pro- and the active form of MMP-9 [21]. In addition, immunohistochemical analysis of estrogen receptor (ER; monoclonal, clone SP1, RTU, sCC1, Ventana Medical Systems), progestrone receptor (PR; monoclonal, clone 1E2, RTU, sCC1, Ventana Medical Systems), HER2 (monoclonal, clone 4B5, RTU, sCC1, Ventana Medical Systems), Ki-67 (monoclonal, clone SP6, dilution 1/100, pretreated sCC1, BioCare medical) were used as surrogate markers of breast cancer molecular subtypes [22]. Antigen retrieval was performed with proprietary reagents followed by incubation with the primary antibody. Sections were then incubated with a specific secondary biotinylated antibody for 32 minutes. Streptavidin horseradish peroxidase, and 3,3-diaminobenzidine were used according to the manufacturer’s instructions (DABmap detection kit, Ventana Medical Systems). Sections were next counterstained with Gill’s hematoxylin and sodium bicarbonate. Finally, each slide was scanned at high resolution (40X) using the Nanozoomer Digital Pathology equipment (Hamamatsu, Bridgewater, NJ). Two independent pathologists reviewed all stained sections on two separate occasions.

Estrogen receptor (ER) and progesterone receptor (PR) status were scored using Allred’s method. In brief, the sum of the proportion and average intensity scores of positive tumor cells were calculated and results displayed on a scale ranging from 0 to 8. The cutoff point used to differentiate between positive and negative samples were as follows: tumors with Allred scores ≥ 3 (corresponding to as few as 1% to 10% weakly positive cells) were considered to be positive. Those tumors that had Allred score of less than 3 were considered to be negative. HER2 overexpression was carried out according to the College of American Pathologists (CAP)-approved scoring system as follows: no immunostaining or membrane staining which is incomplete or barely perceptible within ≤ 10% of the invasive tumor cells → 0; incomplete membrane or barely perceptible staining within >10% of invasive tumor cells → 1+; circumferential membrane staining that is incomplete and/or weak/moderate within >10% of the invasive tumor cells or complete membranous staining that is intense within ≤ 10% of the invasive tumor cells → 2+ and circumferential membranous staining that is complete and intense → 3+ [20]. Scoring of MMP-9 and Ki-67 expression on each core was carried out using a two tier scoring system. The first parameter corresponds to the percentage of immunoreactive cells also known as the quantity score (QS). QS was estimated as follows (no staining was scored as 0, 1-10% of cells with positive staining were scored as 1, >10- 50% as 2, >50-70% as 3, and >70-100% as 4). We next assessed the second parameter (staining intensity score), which was rated as follows: No staining → 0, weak staining →1, moderate staining → 2, and strong staining → 3. The product of the quantity and the staining intensity scores represents the total IHC score that ranges from 0 to 12 [23, 24]. IHC scores of 0 to 4 were considered to represent low levels of expression while score from >4 to 12 were considered as high levels of expression.

IHC staining for ER, PR, HER2 and Ki-67 were used as surrogate markers to classify breast cancer tumors into luminal A, luminal B, HER-2 positive and triple negative breast cancer. Luminal A was defined as being (ER positive, PR positive, HER-2 negative and Ki-67 < 14%), luminal B was defined as being either (ER, PR, HER-2 positive) or (ER positive, PR positive, HER-2 negative and Ki-67 ≥ 14%). Triple negative breast cancers consisted of tumors that lack expression of ER, PR and HER-2. HER-2 positive tumors that failed to express either ER or PR were considered to belong to the HER-2 positive subtype [22].

### Statistical analyses

All statistical analyses were carried out using different packages of the R language (http://www.R-project.org/). The distribution of MMP-9 among different molecular subtypes is depicted using bar charts. Non-parametric tests were used due to the nature of ordinal and categorical data. The overall relationship between MMP-9 scores and molecular subtypes was evaluated using the chi-square test. Correlation analysis for immunohistochemical expression levels was carried out using the Spearman’s rho correlation coefficient. Chi-square test was realized with Yates’ continuity correction and a two-sided Fisher exact test was performed to analyze metastasis. Kaplan-Meier plot was drawn to show the overall survival for low-level and high-level expression of MMP-9. Statistical significance was considered, with a *p*-*value* less than 0.05. Univariate and multivariate logistic regression were used to identify the significant factors among histological grades, histological subtypes, molecular subtypes, metastasis and age that affect the level of MMP-9 expression. The results were interpreted in terms of odds ratio (OR). Univariate and multivariate Cox models were used in survival analysis and the results were interpreted in terms of relative risk (RR). Statistical significance was determined by the confidence interval (CI). Only CI that does not include 1 are considered significant.

## Results

### *In silico*analysis: MMP-9 is overexpressed in basal-like and HER2-positive breast cancers

**Figure 1:**
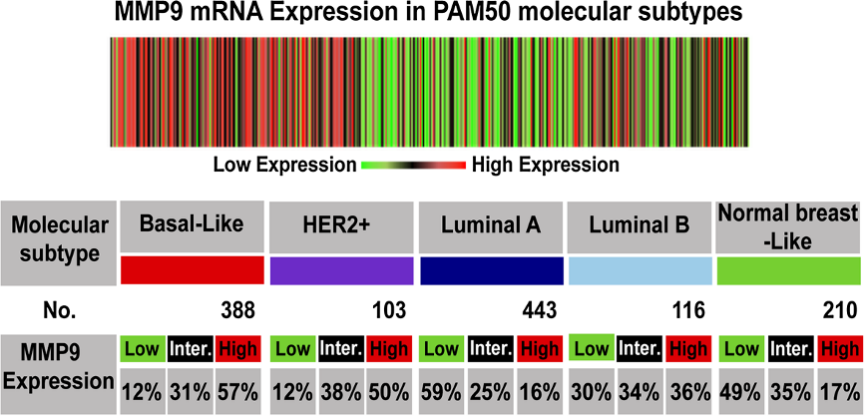
***In silico*** **analysis of** ***MMP*** **-** ***9*** **mRNA expression in breast cancer subtypes.** The heat map and table are produced from the bc-GenExMiner database v3.0 showing the expression of *MMP-9* at mRNA level in different molecular subtypes of breast cancer as determined by PAM50. Overexpression of *MMP-9* is associated with basal-like and HER2-positive breast cancers. The “aov” and “TukeyHSD” functions were carried out to compare the mRNA levels within each breast cancer molecular subtypes.

**Figure 2:**
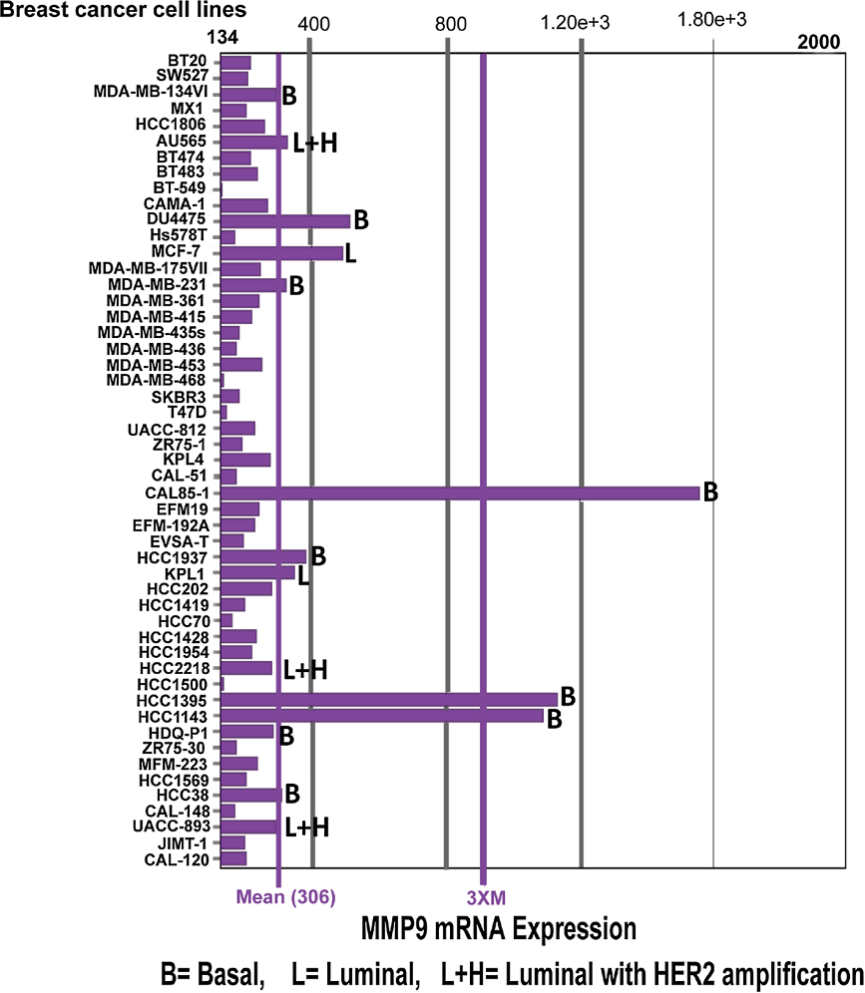
**Expression of** ***MMP-9*** **mRNA in human breast cancer cell lines.** *In silico* analysis showing elevated *MMP-9* mRNA expression levels in basal-like breast cancer cell lines (e.g. CAL85-1, HCC1395, HCC1143, DU4475, HCC1937, MDA-MB-231 and HCC38). Luminal breast cancer cell lines with HER2 amplification also display stronger *MMP-9* mRNA expression (AU565, UAA-893 and HCC2218). MCF7 and KPL1 cell lines are the only luminal cell lines with mildly elevated *MMP-9* mRNA expression. (B = basal, L = luminal, L + H = Luminal with HER2 amplification).

The web application bc-GenExMiner [17] was used to compare the mRNA levels within each breast cancer molecular subtype on a dataset comprising 1210 microarrays. In brief, the gene expression data is given for those patients that could be assigned to a certain molecular subtype (robust classifications for 1210 patients). In Figure 1, the table indicates for each subtype the proportion of patients with low, intermediate, and high gene expression. Gene expression values were being beforehand split in order to form three equal groups. This means that “high expression” is the 1/3 of the patients with highest expression of *MMP*-*9* and “low expression” is the lower 1/3 of the patients. As depicted in Figure 1, 57% of basal-like and 50% of HER2-positive breast cancer patients expressed high levels of *MMP*-*9*. In comparison, only 12% of those subtypes had a reduced expression of *MMP*-*9*. In sharp contrast, only 16% of the luminal A breast cancer subtype demonstrate increased expression of *MMP*-*9*. Data from the luminal B subtype indicate that 36% of patients have high levels of *MMP*-*9* expression while approximately 30% maintained low levels of *MMP*-*9*. To expand on the results obtained from the microarray datasets, we investigated mRNA expression of *MMP*-*9* in 51 breast cancer cell lines of different molecular subtypes [25–27] using publically available microarrays and mRNA sequencing breast cancer cell line datasets [18]. As shown in Figure 2, overexpression of *MMP*-*9* was present in basal-like breast cancer cell lines CAL85-1, HCC1395, HCC1143, DU4475, HCC1937, MDA-MB-231 [28] and HCC38. Interestingly, many luminal breast cancer cell lines known to have HER2 gene amplification (AU565, UAA-893 and HCC2218) also exhibited high levels of *MMP*-*9* expression. Notably, MCF7 and KPL1 cell lines were the only luminal cell lines that revealed a modest increase in *MMP*-*9* expression above baseline levels [29].

### MMP-9 expression is markedly decreased or absent in normal human breast tissue

**Figure 3:**
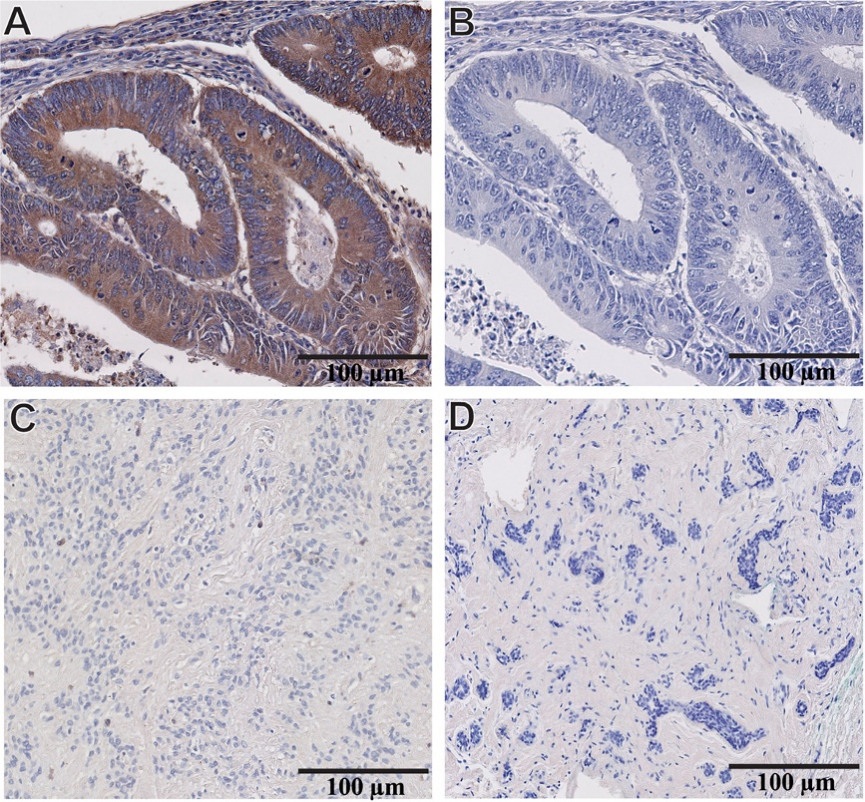
**Validation of MMP-9 antibody specificity for IHC studies. (A)** Human colorectal carcinoma with intense cytoplasmic labeling of the cancer cells after incubating the section with MMP-9 primary antibody. **(B)** Adjacent section from the same colorectal tumor incubated with a non-immune serum that contains IgG (same isotype/ same species) showing complete lack of expression of MMP-9. **(C)** Benign myofibroblastoma of breast tissue and **(D)** Benign breast fibroadenoma do not express MMP-9. Magnification 20X **(A-D).**

Optimization of MMP-9 immune reactivity was a prerequisite to validating the specificity of the IHC reaction. In accordance with the Human Protein Atlas [30] and a review of the literature, human colorectal carcinoma was used as a positive control to assess the levels of MMP-9 expression in human cancers [31]. Our results are in complete agreement with this prediction as shown by the strong cytoplasmic labeling observed in colorectal carcinoma cells (Figure 3A). Additional adjacent sections from the same colonic tumor incubated with a non-immune serum containing IgG (same isotype/same species) remained entirely negative. Of note, all subsequent steps of the immunostaining reaction such as addition of the secondary antibody and the revealing reaction were carried out in a strictly identical fashion (Figure 3B). We also thought fit to include benign breast lesions such as myofibroblastoma (Figure 3C) and fibroadenoma (Figure 3D) as negative controls [20]. Again, no immune reactivity could be detected after the successive addition of MMP-9 primary antibody, secondary antibody and chromogen.

**Figure 4:**
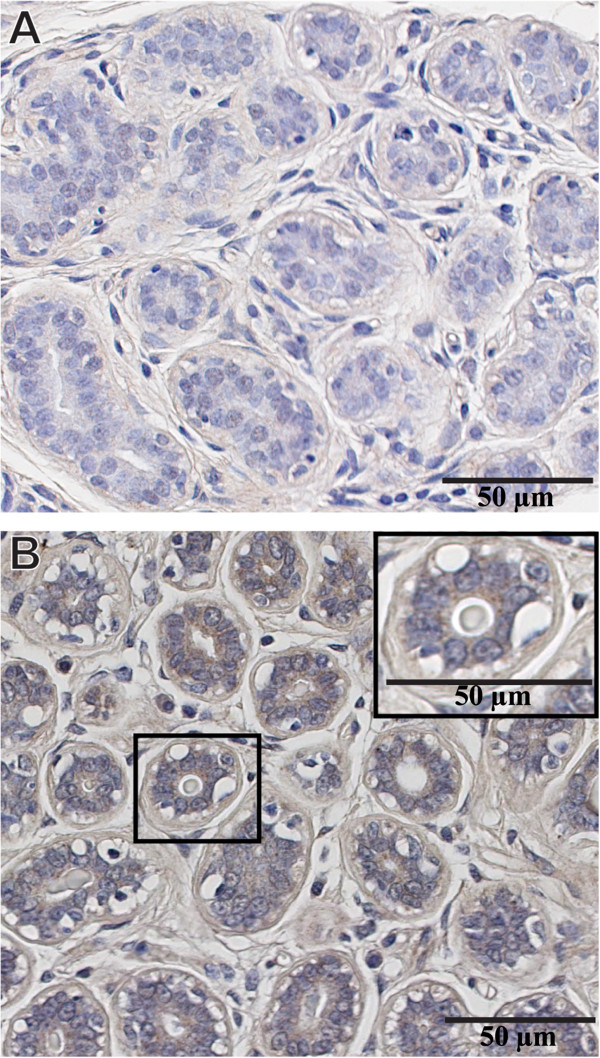
**Expression of MMP-9 in normal breast tissue. (A)** Normal breast lobule lacking MMP-9 expression in both luminal and myoepithelial cells. Adjacent stromal cells also fail to express MMP-9 (74% of the patients). **(B)** Normal breast tissue exhibiting faint expression of MMP-9 in the cytoplasm of luminal cells, myoepithelial cells and in a few stromal cells surrounding normal breast acini. **A** &**B** are two distinct normal breast tissue from the same TMA incubated with anti-MMP9 antibody. Magnification 40X (A&B), 63X inset in Figure 4B.

Once all immunostaining conditions were satisfactorily established, we carried out IHC reactions on TMAs comprising both normal and neoplastic breast tissues. Our results indicate that 74% of normal breast tissues fail to express any MMP-9 reactivity in the luminal, myoepithelial cells and stromal cells surrounding normal breast ducts (Figure 4A). However, in a minority of normal breast tissues (26%) MMP-9 was faintly expressed and restricted to the cytoplasm of luminal, myoepithelial and a few adjacent stromal cells (Figure 4B). MMP-9 did not label either the nucleus nor the cell membrane of any of these cells. Notably, the level of MMP-9 expression in the luminal cells consistently exceeded that present in the adjacent stromal cells.

### Elevated levels of MMP-9 are present in carcinoma cells of triple negative, HER2-positive tumors and nodal metastases

**Figure 5:**
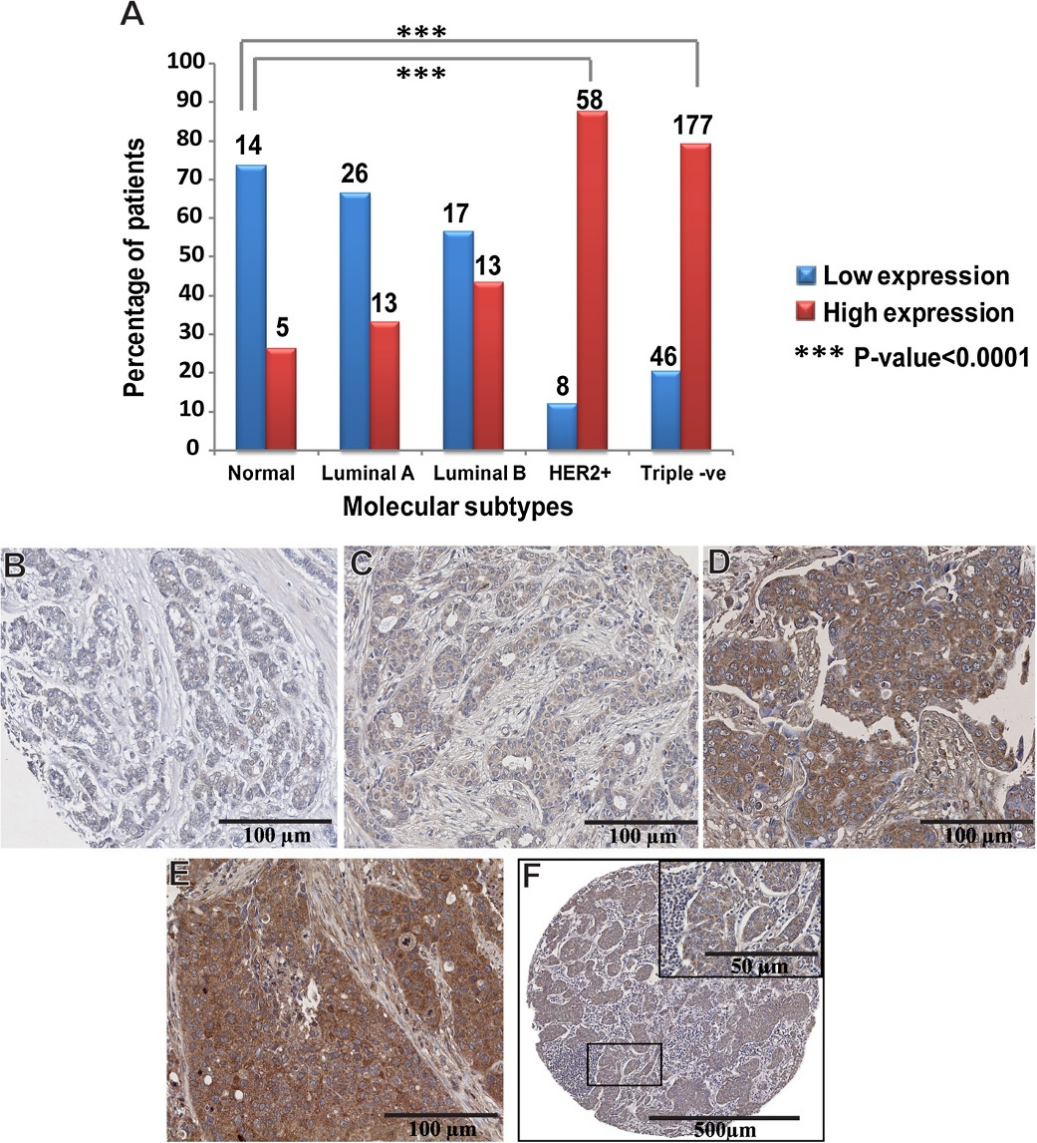
**Overexpression of MMP-9 is associated with triple-negative, HER2-positive breast tumors and nodal metastases. (A)** Histogram showing percentage of breast cancer patients in each molecular subtype category that express low and high level of MMP-9. Both HER2-positive and triple-negative subtypes demonstrate elevated levels of MMP-9 that are significantly different from those observed in normal breast tissue. The number of patients in each group was mentioned over each bar. The overall relationship between MMP-9 scores and molecular subtypes was evaluated using the chi-square test. **(B)** Luminal A and **(C)** Luminal B subtypes showing low level of MMP-9 expression. **(D)** HER2-positive and **(E)** Triple-negative subtypes displaying strong cytoplasmic labeling in cancer cells and surrounding stromal cells. **(F)** Metastatic lymph node demonstrating elevated levels of MMP-9 expression in the cytoplasm of metastatic breast cancer cells. The surrounding lymphocytic and stromal cells did not stain with anti-MMP-9 antibody. Magnification 20X (B-E), 5X (F), 40X inset in Figure 5F.

Next we aimed to validate the results obtained from the *in silico* analysis on human breast tissue. We studied the expression of MMP-9 at the protein level and assessed the cellular and subcellular localization of MMP-9. MMP-9 expression was evaluated in 300 human tumor tissues representative of each molecular subtypes of breast cancer whose definition was based on the use of the following surrogate markers: ER, PR, HER2 and Ki-67 [22]. As shown in Figure 5A, only 33.3% of luminal A (*p* = 0.05) and 43.3% of luminal B (*p* < 0.01) expressed elevated levels of MMP-9. In contrast, high levels of MMP-9 expression were found in 87.9% of HER2-positive and 79.4% of triple-negative breast cancer when compared to normal (*p* < 0.001). Low levels of MMP-9 expression were detected in the cytoplasm of cancer cells in both luminal A and B breast tumors. Indigenous stromal cells surrounding cancer cells in luminal A and B revealed only faint levels of MMP-9 expression (Figure 5B and C). On the other hand, elevated levels of MMP-9 expression were detected in the stroma surrounding cancer cells in both triple-negative and HER2-positive breast cancer. Nevertheless, the level of MMP-9 in the cytoplasm of cancer cells always exceeded that found in adjacent stromal cells (Figure 5D and E). Furthermore, when MMP-9 levels were evaluated in the cytoplasm of carcinoma cells present in 13 metastatic lymph nodes, it was found that all tumor cells (100%) displayed elevated levels of MMP-9 whereas the surrounding lymphocytic and stromal cells failed to express MMP-9 (Figure 5F).

We next conducted univariate logistic-regression analysis on our data to sort out the role of a number of parameters such as histological grades, molecular subtypes and metastasis on the level of MMP-9 expression. This analysis confirmed the association between the high levels of MMP-9 expression (total scores >4) with tumors of high histological grade (Grade III) including both HER2-positive and triple-negative breast cancers (Table 1). Hence, we can safely conclude that MMP-9 protein expression *in vivo* strongly supports both *in silico* analyses on microarray dataset as well as data gathered from analysis of breast cancer cell lines.

**Table 1:** **Univariate analysis of different factors that could affect level of MMP-9 expression**

Parameters	OR	95% CI	***p-value***
Grades			
Grade I	Reference		
Grade II	1.74	0.82-3.73	0.15
Grade III	2.61	1.36-5.08	*< 0.001*
Molecular subtypes			
Luminal A	Reference		
Luminal B	0.51	0.26-0.99	0.05
HER2-positive	8.01	3.85-18.46	*0.001*
Triple-negative	3.90	2.48-6.19	*0.001*
Metastasis (No)	Reference		
Metastasis (Yes)	2.17	1.48-3.23	*0.001*

OR = odds ratio, CI = confidence interval.

### Overexpression of MMP-9 is associated with a higher incidence of metastases

**Figure 6:**
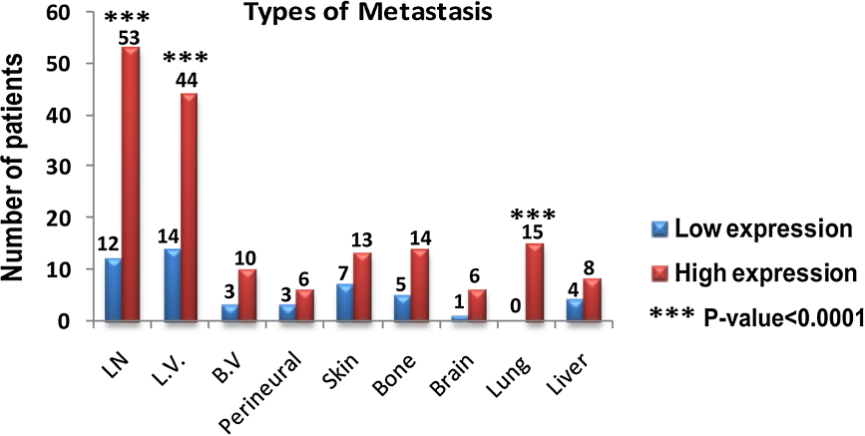
**Overexpression of MMP-9 is associated with a higher incidence of metastases.** Increased expression of MMP-9 is associated with higher incidence of metastasis. Only lymph node, lymphovascular invasion and lung metastases reached the level of statistical significance when compared to patients with low MMP-9 expression. Chi-square test was realized with Yates’ continuity correction and a two-sided Fisher exact test were performed to analyze metastases.

We next investigated whether elevated levels of MMP-9 protein expression in carcinoma cells could predict the occurrence of metastases, relapse and poor survival rates. To that end, we reviewed the clinical charts of 200 patients for the period extending from 2000 to 2013. Out of 200 Patients, 121 (60.5%) patients have high MMP-9 expression and 79 (39.5%) pateints have low MMP-9 expression. Increased levels of MMP-9 were found to be associated with a higher incidence of metastasis (Figure 6). The results were considered significant when the percentage of patients who developed metastases significantly differed in terms of low and high levels of MMP-9 expression. Only lymph node (*p* < 0.001), lymphovascular invasion (*p* = 0.007) and lung metastasis (*p* = 0.001) reached statistical significance when compared to patients with low MMP-9 expression. Additional file 1 indicates the distribution of high and low MMP-9 expression in patients with and without metastases.

Univariate analysis of our data demonstrated the association between elevated levels of MMP-9 expression and the increased likelihood to develop metastasis (OR = 2.17, 95%CI = 1.48-3.23, p-value = 0.0001) (Table 1). Moreover, to examine which clinical factors could affect the relationship between MMP-9 and metastasis, multivariate logistic-regression analysis was carried out. Triple-negative molecular subtype proved to be the only statistically independent predictor of metastasis (OR = 7.92, 95%CI = 2.90-21.6, *p*-*value* =0.0001) (Table 2). This suggests that triple negative breast cancer have a stronger clinical value in predicting metastasis rather than any of the other biological factors examined.

**Table 2:** **Multivariate analysis model of MMP-9 that include metastasis, histological subtypes and molecular subtypes**

Parameters	OR	95% CI	***p value***
Metastasis			
Luminal A	0.97	0.45-2.07	0.93
Luminal B	3.52	0.81-15.27	0.12
HER2-positive	0.77	0.16-3.61	0.79
Triple-negative	7.92	2.90-21.61	*0.001*

OR = odds ratio, CI = confidence interval.

### High levels of MMP-9 are associated with a shorter latency to relapse and shorter survival after relapse (SAR)

**Figure 7:**
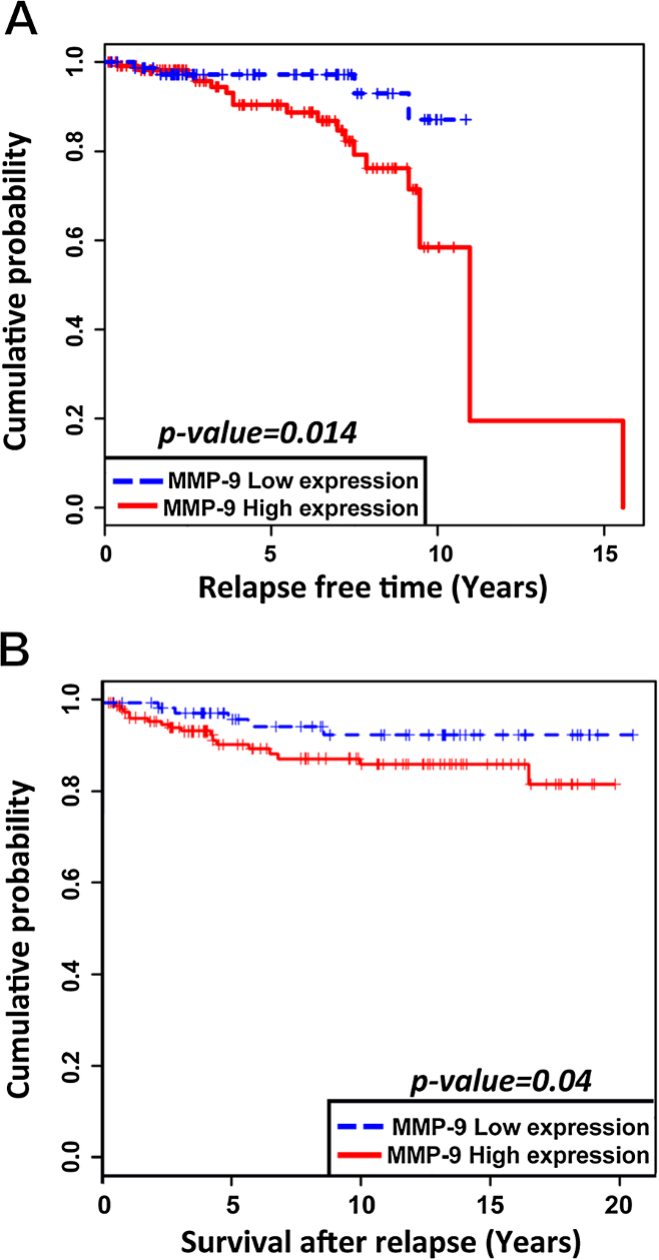
**Overexpression of MMP-9 is associated with shorter time to relapse and shorter survival after relapse. (A)** High levels of MMP-9 expression are associated with shorter time to relapse (*p = 0.014*). **(B)** High levels of MMP-9 expression are associated with shorter survival after relapse (*p = 0.04*).

Likewise, when we looked at the association between MMP-9 and relapse, we found that enhanced expression of MMP-9 was associated with a shorter latency to clinical relapse: (Mean time for relapse = 3912 days, n = 121) which is statistically significant (*p* = 0.014). This contrasts with the values observed in patients with low MMP-9 levels of expression (Mean time for relapse = 4957 days, n = 79) (Figure 7A). However, using a multivariate analysis, histological grades, histological subtypes and molecular subtypes were found to have no impact on relapse in this patient’s population.

Finally, the Kaplan-Meier overall survival (OS) curve obtained from the same cohort of patients indicates that increased expression levels of MMP-9 are associated with a shorter OS (Mean OS = 6469 days, n = 16) when compared to those tumors expressing low levels of MMP-9 (Mean OS = 6984 days, n = 6). However, no significant difference could be identified between OS for patients having high or low levels of MMP-9 expression. Interestingly, univariate analysis shows that patients with higher levels of MMP-9 expression harbor a significant high risk of death after relapse (RR = 3.05, *p* = 0.04) (Figure 7B). It is also worth mentioning that we could not find any statistically significant correlation between the expression of MMP-9 in the tumor stroma and the occurrence of metastasis or overall survival in the same patients.

## Discussion

In the present paper, we provide both indirect and direct evidence that MMP-9 participates to breast cancer progression and impact on clinical outcome. There are many studies reporting the association of elevated levels of MMP-9 with a higher incidence of metastases and poor clinical outcome. We found that high expression of MMP-9 is specifically correlated with high-grade breast cancers that include both triple-negative and HER-2 positive breast cancers.

Previous studies have provided conclusive evidence that MMP-9 is involved in several key processes that contribute to breast cancer development, progression, invasion and metastasis [32–34]. Here we performed *in silico* analysis of 1210 DNA microarrays of human breast cancer tissues and RNA sequencing data of 51 human breast cancer cell lines to assess *MMP*-*9* mRNA expression. We found that *MMP*-*9* mRNA expression in both basal-like and HER2-positive tumors reached significantly higher levels than those observed in the luminal A category. When the expression of *MMP*-*9* in breast cancer cell lines is considered, it is worth mentioning that cell lines with a basal-like phenotype and those that overexpressed HER2 reached the highest levels of *MMP*-*9* expression. In contrast, cell lines with luminal phenotype failed to demonstrate elevated levels of *MMP*-*9*. This strongly suggested to us that MMP-9 expression varied according to cell differentiation and histological grades. Hence, we decided to construct human breast cancer tissue microarrays (TMA) comprising a wide selection of tumors belonging to each category of breast cancer molecular subtypes. Those tumors were classified as triple-negative, HER2-enriched, luminal A and luminal B based on the expression profile of four surrogate markers (ER, PR, HER2, Ki-67) [22]. We also included normal breast tissue to serve as a basis for comparison. To thoroughly validate the robustness of our IHC assay we first included a number of internal and external controls. Whereas colonic adenocarcinoma strongly expressed MMP-9, two benign breast lesions (fibroadenoma and myofibroblastoma) failed entirely to express MMP-9 under the same conditions. Once the experimental procedures were set up, we performed the IHC assay on TMAs. One important finding was that normal breast tissue displayed either a complete lack of positivity or barely perceptible labeling with the antibody directed against MMP-9. This is consistent with previous observation by others reporting only a weak expression of MMP-9 in normal breast tissue [35, 36]. Indeed, low levels of MMP-9 expression in normal breast tissue are expected since in most tissues MMP-9 is an inducible and not a constitutively expressed gene [37]. Evidently, this sharply contrasts with the high levels of expression of MMP-9 found in the cytoplasm of both HER2-positive and triple-negative breast cancers cells. Hence, our findings support the conclusions of recently published studies indicating a positive correlation between high levels of MMP-9 expression and triple-negative breast cancers [20, 38, 39]. Our results may also explain the findings of La Rocca et al. who showed that high serum levels of MMP-9 are present in HER2 amplified breast cancers [40]. In this context, abnormally elevated levels of MMP-9 can be envisaged as a response to local secretion of inflammatory cytokines and growth factors, such as interleukin 1 (IL-1) and tumor necrosis alpha (TNFα), which may lead to either activation of NF-kB, a well-known inducer of MMP-9 production, or hypomethylation of its promoter [41]. One cautionary note should be raised though, since high levels of MMP-9 do not necessarily imply high MMP-9 activity as the protein is produced as an inactive pro-enzyme. Moreover, active MMP-9 can be completely neutralized by protease inhibitors such as tissue inhibitors of metalloproteinases (TIMPs) [42]. As for the production site of MMP-9 in breast tumors, our results suggest that carcinoma cells are the main source of MMP-9 given that adjacent stromal cell consistently exhibited a much weaker degree of expression.

Finally, we wanted to correlate clinical outcome characteristics such as onset of metastasis, survival rates and tumor relapse with MMP-9 levels. Our results confirm that overexpression of MMP-9 is tightly correlated with lymphovascular invasion, regional node metastasis, a shorter time to relapse and a reduced SAR. Taken together, our data underscore the role of MMP-9 in promoting breast cancer metastases in lymph node and lungs. This finding is consistent with both *in vitro and in vivo* studies reporting high levels of MMP-9 expression in highly metastatic cell lines [43] and its contribution in metastatic progression [39]. Also, this supports the finding of van ’t Veer et al. [44] who demonstrated in a DNA microarray study that MMP-9 is significantly upregulated in poor prognosis signature of breast cancer. Although we have not directly addressed the question on how MMP-9 fosters invasion and nodal metastasis, there are numerous conceivable explanations that can be put forth such as alteration of basal membrane components, diminished cell-to-cell adhesion, release of ECM-bound growth factors and chemotactic molecules, stimulation of angiogenesis and induction of the epithelial-mesenchymal transition (EMT) [45–49].

At any rate, our findings clearly emphasized the clinical potential of MMP-9 as a prognostic biomarker in breast cancer. This is in agreement with Wu et al. [50] who suggested the potential role of MMP-9 as a biomarker for breast cancer progression. Interestingly, the first fully commercialized and FDA approved microarray-based multigene assay for breast cancer, MammaPrint®, does include MMP-9 among its 50 panel genes [51, 52]. Given on the one hand the overwhelming interest in developing prognostic and predictive breast cancer assays and, on the other, the recognition that so called “wound-healing” or “invasion” gene signatures are important to predict tumor relapse and benefit to chemotherapy, one might consider including MMP-9 alone or in combination with other genes in the development of other multigene multiplex assays.

## Conclusion

In summary, our results indicate that overexpression of MMP-9 is closely associated with breast cancers of high histological grade including triple-negative and HER2-positive molecular subtypes. Increased levels of expression of MMP-9 are also correlated with the onset of nodal metastases, a reduced time interval to relapse and a shorter SAR. Taken together, our findings suggest that the differential expression of MMP-9 contributes to breast cancer heterogeneity and is a key characteristic of the “molecular signature” of subsets of breast cancer. In our opinion, MMP-9 expression could help segregate subsets of aggressive breast cancer into clinically meaningful subtypes. Lastly, our results suggest that MMP-9 is a valuable gene/protein candidate to be considered in the development of a multi-gene panel or multiplex proteomic assay to predict clinical outcome.

## Electronic supplementary material

Additional file 1: **Number of patients with or without metastasis associated with either high or low MMP-9 expression.** (PDF 45 KB)

## Abbreviations

CAP: College of American Pathologists
CI: Confidence interval
DNA: Deoxyribonucleic acid
ECM: Extracellular matrix
EMT: Epithelial-mesenchymal transition
ER: Estrogen receptor
FDA: Food and drug administration
FFPE: Formalin-fixed, paraffin-embedded
HER2: Human Epidermal Growth Factor Receptor 2
H&E: Haematoxylin and eosin
IgG: Immunoglobulin G
IHC: Immunohistochemistry
IL-1: Interleukin 1
mRNA: Messenger ribonucleic acid
MMPs: Matrix metalloproteinases
MMP-1: Matrix metalloproteinase-1
MMP-8: Matrix metalloproteinase-8
MMP-13: Matrix metalloproteinase-13
MMP-9: Matrix metalloproteinase-9
NF-kB: Nuclear factor kappa-light-chain-enhancer of activated B cells
OR: Odds ratio
OS: Overall survival
PR: Progesterone receptor
QS: Quantity score
RNA: Ribonucleic acid
RR: Relative risk
SAR: Survival after relapse
SBR-EE: Scarff-bloom-richardson-ellis-elston
TIMPs: Tissue inhibitors of metalloproteinases
TMA: Tissue microarray
TNFα: Tumor necrosis factor alpha.
