# Supplementary material for: MMP-9 expression varies according to molecular subtypes of breast cancer
Source: BMC Cancer. 2014 Aug 23;14:609. doi: 10.1186/1471-2407-14-609 (PMC4150970; doi:10.1186/1471-2407-14-609)
Supplement: Supplementary file 1 — Additional file 1: Number of patients with or without metastasis associated with either high or low MMP-9 expression. (PDF 45 KB) [file 12885_2014_4789_MOESM1_ESM.pdf]

Additional file 1

Number of patients with or without metastasis associated with either high or low MMP-9 expression.

	Metastasis	High Expression	Low Expression
Lymph node (L.N.)	Yes	53	12
	No	68	67
Lympho-vascular (L.V.)	Yes	44	14
	No	77	65
Blood vessels (B.V.)	Yes	10	3
	No	111	76
Perineural	Yes	6	3
	No	115	76
Skin	Yes	13	7
	No	108	72
Bone	Yes	14	5
	No	107	74

Brain	Yes	6	1
	No	115	78
Lung	Yes	15	0
	No	106	79
Liver	Yes	8	4
	No	113	75
